# The Good, the Bad, and the Ugly: in search of gold standards for assessing functional genetic screen quality

**DOI:** 10.15252/msb.20145372

**Published:** 2014-07-01

**Authors:** Bastiaan Evers, Rene Bernards, Roderick L Beijersbergen

**Affiliations:** Division of Molecular Carcinogenesis and Cancer Genomics Centre Netherlands, The Netherlands Cancer InstituteAmsterdam, The Netherlands. E-mail: r.beijersbergen@nki.nl

## Abstract

Variable screen quality, off-target effects, and unclear false discovery rates often hamper large-scale functional genomic screens in mammalian cells. Hart *et al* (2014) introduce gold standard reference sets of essential and non-essential genes, aiming at standardizing the analysis of genome-wide screens. This work provides a framework to compare both the quality and analysis methods of functional genetic screens.

In the last decade, several screening technologies have been developed that allow for genome-scale perturbation of gene expression in mammalian cells. These include siRNA, shRNA, gene traps, and more recently CRISPR-based gene editing technologies. In particular, large-scale shRNA screens have been applied broadly to identify genes that are lethal under specific circumstances, for example in combination with a drug treatment or in the context of disease-specific genetic alterations. These context-specific essential genes could represent interesting new therapeutic targets. Unfortunately, results from such large-scale screens are often met with limited reproducibility and sensitivity, due to extensive off-target effects and variable knockdown efficiency. In addition, several analytical methods with different criteria for hit selection are in use, further complicating the interpretation and comparison of various screening efforts. The identification of a set of context-independent essential and non-essential genes would be a great asset in the evaluation of these technologies and the accompanying analytical tools. Hart *et al* (2014) developed such references and used them to develop a quality assessment and analysis framework that can be applied widely to functional genomic screens. The use of these tools should improve the performance and ability to compare genetic screens and thereby increase their potential to uncover novel biologic insights and new treatment strategies.

The authors assembled standard sets of essential and non-essential genes based on the analysis of a previously published collection of genome-scale shRNA screens for 72 human cancer cell lines (Marcotte *et al*, 2012). First, a seed set of essential genes, showing consistent anti-proliferative effects across the panel of cancer cell lines, was defined. This list was filtered for those genes that show constitutive and invariable expression, arguably characteristics of essential genes. On the other hand, a reference list of non-essential genes was generated by selecting protein-coding genes that show invariably low or absent expression. These gene sets were used to train a Bayesian classifier for gene essentiality. Every individual screen was then analyzed to classify genes as either essential or as non-essential. An *F*-measure, essentially a metric of the quality of a screen, was calculated based on recall and precision of a left-out test set. Finally, a “core essentials” list of 291 genes was generated by selecting genes that are essential in more than half of the high-quality screens (*F*-measure ≥ 0.75) (Fig1). A more loosely defined “total essentials” list of 823 genes was constructed using a modeling approach that estimated the FDR of this list to be 6–11%.

Analysis of the “core essential” genes shows that while their mouse or yeast orthologs are often also essential, they are less likely to have human paralogs that could act redundantly. Interestingly, when all essential mouse genes are split between those that have human orthologs in the “core essentials” list and those that do not, an enrichment of disease genes is observed only in the latter, “peripheral essentials”. Perhaps the “core essentials” represent genes that upon loss are completely incompatible with cell survival, while the “peripheral essentials” genes are only necessary for certain organismal or developmental aspects. This would predict that life is less tolerant to mutations in “core essentials” than in “peripheral essentials”, a theory indeed supported when analyzing a large set of published human sequenced exomes.

**Figure 1:**
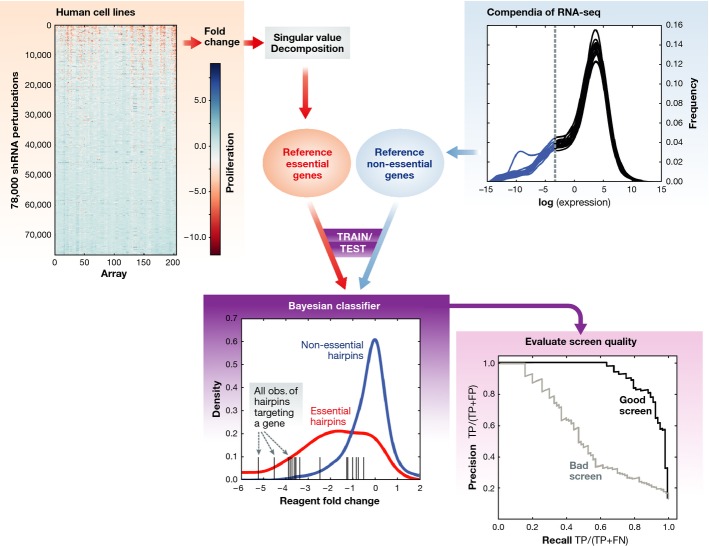
Reference sets of essential and non-essential genes were assembled based on the analysis of pooled genome-scale shRNA screens across a set of 34 human cancer cell lines (Marcotte *et al*, 2012) These reference sets were used to train a Bayesian classifier for gene essentiality, developed to evaluate whether the distribution of fold-changes for hairpins targeting a given gene better matched the distribution of fold-changes of hairpins targeting training sets of essential or non-essential genes. Every individual screen was then analyzed to classify genes as either essential or as non-essential. The genes were ranked by Bayes factor, and a precision versus recall (PR) curve was calculated.

Besides comparison of datasets, the presented Bayesian approach also allows for the evaluation of different data analysis methods. Compared to two often-used algorithms, the method of Hart *et al* (2014) performs better in identifying essential genes in a CAPAN-2 cell line screen. The performance is even further improved when gene expression information is included in the algorithm, assuming a positive correlation between expression levels and gene essentiality.

The *F*-measure as a screen performance metric allows not only for quality assessment of a single screen, but upon simultaneous analysis of many screens, it can also reveal factors that may influence RNAi screening quality. In this way, it was observed that the expression of *AGO2*, a core component of the RNAi machinery, correlates with screen quality. Indeed, it was recently shown that *AGO2* overexpression can enhance RNAi and is thus an interesting approach to improve on poorly performing shRNA screens (Börner *et al*, 2013).

Another factor affecting false-negative rates in screens was uncovered when a negative correlation was detected between copy number and the ability to identify an essential gene. A tempting explanation for this is that the higher expression levels resulting from the amplification make it more difficult to fully knock down the gene expression by RNAi perturbation. A possible solution to this issue is the use of CRISPR technologies, which in principle have the potential to fully knock out any given gene. It should be noted, however, that the penetrance of such events in screening efforts is not 100% and that CRISPR technology also suffers from off-target effects. Nevertheless, a first analysis, by Hart *et al* (2014) using their framework of essential and non-essential genes, suggests that CRISPR screens have a greater sensitivity than shRNA screens, although false discovery rates are non-trivial using this technology.

Hart *et al* (2014) have done an excellent job in creating lists of essential and non-essential genes. The degree to which any screen identifies these “core essentials” can be used as a measure of its accuracy but also for standardization and hit selection criteria. This could certainly improve the value and interpretation of large-scale genetic screens. This would be further enhanced if scientists would release along with their published studies, their complete screening datasets for public use. Whether the gene lists presented by Hart *et al* (2014) are indeed gold standards remains to be determined. However, the Bayesian approach taken here can be applied to any dataset and contribute to iterative refinements of the presented lists and thus hold gold for the further improvement of screening technologies.
